# Determining the strength of evidence for an association between sexual indicators and risk of acquiring HIV and sexually transmitted infections: Providing evidence for blood donation policy change

**DOI:** 10.1111/tme.13062

**Published:** 2024-07-22

**Authors:** Joe Flannagan, Katy L. Davison, Claire Reynolds, Susan R. Brailsford

**Affiliations:** ^1^ UK Health Security Agency London UK; ^2^ NHS Blood and Transplant Bristol UK

**Keywords:** blood donation, donor selection, health policy, HIV, STI, transfusion

## Abstract

In 2019 the For The Assessment Of Individualised Risk (FAIR) project began a review of UK blood donor selection policy to determine if a more individualised approach to donor selection could be safely implemented. An evidence base was required to inform selection policy to move from a population to a more individual based policy, specifically what sexual behaviours/indicators should be considered as screening questions to maintain the safety of the blood supply. Eight sexual behaviours/indicators were reviewed: history of bacterial sexually transmitted infections (STIs), chemsex, number of recent partners, condom use, type of sex, sexual health service (SHS) attendance, new sexual partner and exclusivity. We conducted searches in multiple databases to identify literature looking at the association between these behaviours/indicators and HIV/STI acquisition risk. A scoring system to determine strength of evidence was devised and applied to papers that passed screening. Key studies were identified which achieved the maximum score and more in‐depth reviews were conducted for these. We identified 58 studies, including 17 key studies. Strong evidence was found linking a previous bacterial STI, chemsex and increasing numbers of sexual partners to acquisition risk. Condom use, type of sex and new partners were found to have some strength of evidence for this link. SHS attendance and exclusivity had minimal evidence. We recommended that the behaviours/indicators viewed as having strong or some strength of evidence should be considered as screening questions in a more individualised approach to donor selection criteria.

## 1 INTRODUCTION

All four UK blood services have processes in place to minimise the chance of transfusion‐transmitted infections (TTIs), these include donor selection, donation testing and processing, and blood component storage.^1^ In the UK all blood donations are tested for markers of bloodborne infections including HIV, Hepatitis B Virus (HBV), Hepatitis C Virus (HCV) and syphilis, however, the window period of the tests means that very recently acquired infections may not be detected.^2^ Donor selection criteria therefore provide an important blood donation safety measure to reduce the chance that someone with a recently acquired infection would donate. It also provides a safety measure against bloodborne infections which are not currently part of routine tests. Until recently in the UK most donor selection criteria were based on population‐based risks of acquiring a blood borne infection, for example all gay, bisexual or other men who have sex with men (GBMSM) were deferred from donating if they had had sex with another man in the preceding 3 months. This time period was based on the window period for tests conducted. Similar deferral criteria were applied to people in other groups at increased risk of HIV or STIs including people who have been paid for sex, people who had partners with a known infection and people who have had sex with someone from an area with high HIV prevalence, mainly Sub‐Saharan Africa.

In 2019 the For The Assessment Of Individualised Risk (FAIR) project was set up. The aim of this work was to evaluate if it was possible to safely change the blood donation criteria to a more individualised risk‐based approach. This required identifying the best questions to ask people to select lower‐risk individuals to donate, some of whom had been previously deferred from blood donation, while still maintaining the safety of the blood supply. The approach to do this combined epidemiological, behavioural and psychological evidence about current and potential future donors. The FAIR project led to changes in UK blood donation criteria in 2021. This study was conducted as part of the project and contributed to the evidence base to support this change, alongside other evidence relating to these behaviours/indicators regarding recall, acceptability, practicality and more. Many other countries have since made similar changes including Canada, USA and France whereby donor selection processes no longer ask questions about sex between men.

Certain sexual behaviours, or certain indicators of sexual behaviours, are associated with an increased likelihood of acquiring HIV/STIs. Here, we build on work carried out in 2018 at Canadian Blood Services and Héma‐Québec, which asked donors about the frequency of specific sexual behaviours/indicators and to what degree they would be comfortable responding to questions on these as part of their pre‐donation screening. From this, we considered chemsex, number of recent partners, condom use, type of sex, new sexual partner and exclusivity to be of most relevance to a UK donor selection policy. The use of internet/social media to find partners was part of the Canadian work but following discussions with HIV/STI surveillance experts this was not deemed to be a relevant behaviour/indicator or an acceptable avenue of questioning in a UK setting. Following discussions with experts in sexual health we also included two additional behaviours/indicators (history of bacterial STI and Sexual Health Service (SHS) attendance) as they are often considered risk indicators for acquisition of HIV/STIs within UK sexual health settings. Overall, the behaviours/indicators considered to be relevant for selecting donors at lower risk of acquiring infections through sex were: chemsex, number of recent partners, history of bacterial STIs, SHS attendance, new sexual partner, condom usage, exclusivity and type of sex.

Chemsex is a term for the use of drugs before/during sexual activity to facilitate it and it has been linked to participation in sexual practices which increase acquisition risk, such as condomless sex and high numbers of partners.^3^, ^4^ While history of bacterial STIs and attending SHS do not themselves increase acquisition risk it has been suggested they are predictors of future acquisition risk; these are sometimes referred to as proxy measures or indicators of risk.^5^ Condoms act as a barrier to infection during penetrative sex and so their consistent use decreases acquisition risk. Different types of sex carry varying risk of acquisition and so when referring to type of sex in this study we are referring to the variation in acquisition risk between sex acts encompassing oral, vaginal, anal and receptive versus penetrative sex.^6^ While there is no evidence of gonorrhoea nor chlamydia transmission via transfusion, and transfusion‐transmitted syphilis has not been identified in the UK setting since surveillance began in 1996 this review will consider them because they are STIs, which can indicate higher risk sexual practices.^7^ It should be noted that currently antibody tests are used to detect syphilis infection in blood donors, meaning anyone with a history of infection is deferred.

While the association with these behaviours/indicators and HIV/STI acquisition risk is somewhat established, determining the strength of evidence will help to support the formulation of questions to blood donors for a more individualised risk assessment. Important details about the behaviours/indicators from each study were also recorded to provide a finer level of granularity in the evidence to further refine the questions to consider for the more individualised approach.

## 2 MATERIALS AND METHODS

In total five literature searches were conducted in November 2019 in both Medline and EMCare using synonymous terms for HIV, STI and for specific STIs plus terms for the eight sexual behaviours/indicators; chemsex, number of recent partners, history of bacterial STIs, SHS attendance, new sexual partner, condom usage, exclusivity and type of sex (Data S1). To help ensure similarity of the epidemiology in the UK at the time inclusion criteria included literature published after January 1981 and studies conducted in Europe, North America, Australia and New Zealand. In order to align the evidence base more closely with UK blood donors, studies were excluded if their study population included: people under 17 years old, known HIV positive individuals, PrEP users or injecting drug users. Studies on incarcerated populations were also excluded as collection no longer occurs in prisons in the UK. Other exclusion criteria included studies with less than 50 participants, those with no comparison group, co‐infection studies, and those where the risk was not specified, for example people labelled simply as high risk. Studies were screened and included if they assessed the association between at least one of the eight sexual behaviours/indicators and risk of HIV/STI acquisition risk and did not meet any exclusion criteria.

Three rounds of screening were conducted against the aforementioned inclusion/exclusion criteria. The first was by a literature search expert based on title and abstract followed by deduplication by this same expert. The second round was conducted by an epidemiologist also based on title and abstract only and the third screening round was conducted by the same epidemiologist based on the full text. All studies that passed these three screening rounds were included in the analysis.

We wanted to assess the strength of evidence for the link between HIV/STI acquisition risk and each sexual behaviour/indicator based on the literature identified. To do this a scoring system was devised to, after screening, assign a score to each identified study based on the size of the study population and number of study sites (Table 1). The system devised for this was adapted from a similar system used previously by Brady et al.^8^ Any study scoring the maximum of three was classified as a key study. Once all studies had a score, the sexual behaviours/indicators they found linked to acquisition risk were listed next to each study. The score of the study was then applied to the sexual behaviours/indicators listed next to them. So, if a study scored two and found evidence linking acquisition risk with both condom use and number of recent partners then these behaviours both got two points. The scores were added up by sexual behaviour/indicator to reach a final tally. Thereby if four studies which scored one point and two studies which scored three points linked chemsex to HIV/STI acquisition risk the final tally for chemsex was 10 (4 × 1 + 2 × 3).

**TABLE 1:** Scoring system for included studies.

Score	Criteria
1	Study population of 50–199
	One study location & study population of 200–999
2	Multiple study locations & study population of 200–999
	One study location & study population of 1000–4999
3 (key study)	Multiple study locations & study population of 1000–4999
	Study population of 5000+

As well as assigning scores to the behaviours/indicators in each of the identified studies any key findings relating to the sexual behaviours being investigated, which were potentially relevant to a more individualised risk assessment were recorded. This included exact definitions of the sexual behaviours, any time periods applied, and problems encountered when collecting information.

## 3 RESULTS

### 3.1 Number and characteristics of included studies

**FIGURE 1:**
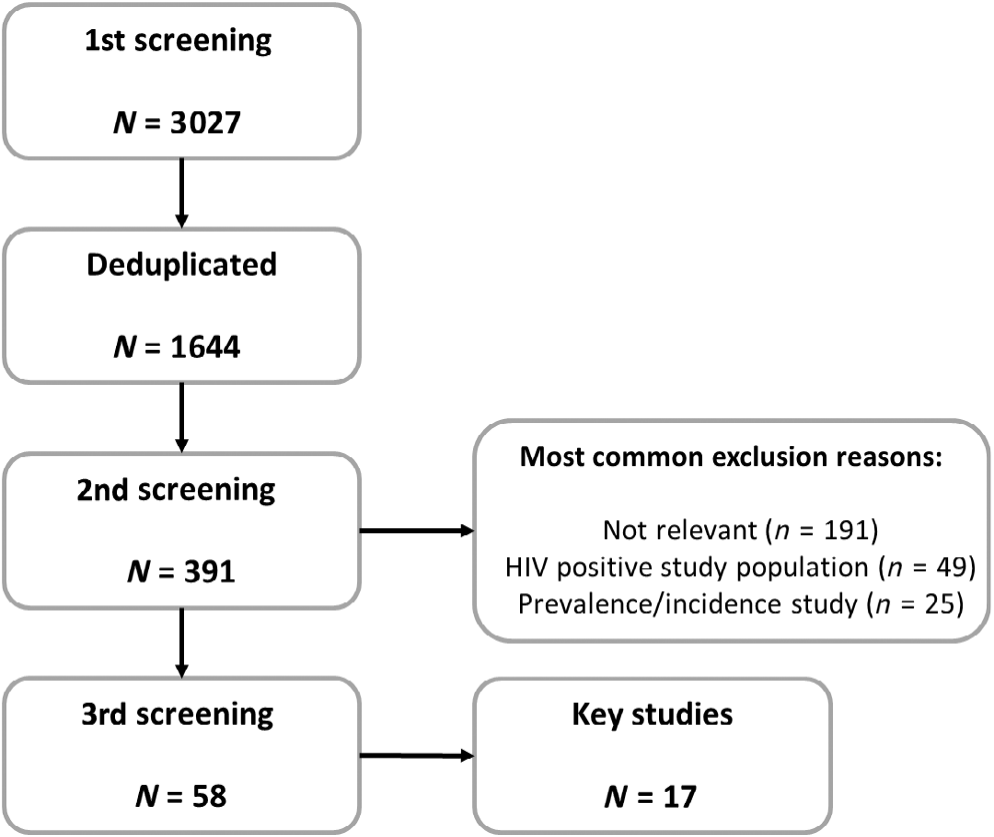
A flow chart to show the screening process for literature.

The result from the screening process can be seen in Figure 1. Fifty‐eight studies were identified and included in subsequent analysis of which 17 were key studies (Table 2). Out of the total nine were case–controls, 24 were cohort, and 25 were cross‐sectional studies with 46 of 58 focused solely on GBMSM populations (Tables 2 and 3).

**TABLE 2:** Details of key studies identified.

Study	Publication year	Study type	Population size	Population description	Country	Chemsex	No. of partners	Previous bacterial STI	GUM attendance	New sex partner	Condom use	Exclusivity	Type of sex
Kassler et al.^9^	1994	Case–control	6175	GUM clinic attendees	USA			X					
Parazzini et al.^10^	1995	Case–control	1711	GUM clinic attendees	Italy						X		
Buchbinder et al.^11^	2014	Cohort	1248	MSM & trans women	USA						X		X
Ackers et al.^12^	2012	Cohort	4684	High‐risk MSM	USA	X	X	X			X		X
Llata et al.^13^	2018	Cohort	14 824	MSM GUM clinic attendees	USA			X					
Desai et al.^14^	2017	Cohort	26 200	MSM GUM clinic attendees	England			X					
Desai et al.^15^	2018	Cohort	1278	MSM GUM clinic attendees	England		X	X					
Katz et al.^16^	2016	Cohort	3715	MSM	USA	X							
Koblin et al.^17^	2006	Cohort	4295	MSM	USA	X	X	X			X		X
Aghaizu et al.^18^	2016	Cross‐sectional	10 364	MSM	England		X	X					
Fournet et al.^19^	2016	Cross‐sectional	3053	Male sex workers	Netherlands			X					
Dickson et al.^20^	2015	Cross‐sectional	3138	MSM	New Zealand		X				X		X
Dukers‐Muijrers et al.^21^	2009	Cross‐sectional	12 949	GUM clinic attendees	Netherlands				X				
Rudy et al.^22^	2009	Cross‐sectional	6435	MSM GUM clinic attendees	USA	X							
Sewell et al.^23^	2017	Cross‐sectional	1484	MSM GUM clinic attendees	UK	X							
Ferrer et al.^24^	2015	Cross‐sectional	2197	MSM	Europe	X		X					
Kohli et al.^25^	2019	Cross‐sectional	16 065	MSM	UK	X							

**TABLE 3:** Details of non‐key studies identified.

Study	Publication year	Study type	Population size	Population description	Country	Chemsex	No. of partners	Previous bacterial STI	GUM attendance	New sex partner	Condom use	Exclusivity	Type of sex
Brewer et al.^26^	2006	Cross‐sectional	241	MSM	USA	X	X	X					
Wagstaff et al.^27^	1999	Cohort	562	African‐American males	USA			X					
Kelley et al.^28^	2015	Cohort	562	MSM	USA			X					
Pathela et al.^29^	2013	Cohort	276	MSM GUM clinic attendees	USA			X					
Turner et al.^30^	2013	Cross‐sectional	326	MSM GUM clinic attendees	USA								X
Zetola et al.^31^	2009	Case–control	13 662	MSM	USA			X					
Suligoi et al.^32^	2002	Cross‐sectional	776	GUM clinic attendees	Italy			X					
Vall et al.^33^	2001	Cross‐sectional	1093	GUM clinic attendees	Spain			X					
Glynn et al.^34^	2017	Cohort	871	MSM GUM clinic attendees	USA								X
Cheung et al.^35^	2016	Cohort	5256	MSM GUM clinic attendees	Australia			X			X		
Kim et al.^36^	2003	Cross‐sectional	564	MSM GUM clinic attendees	USA	X							
Donovan et al.^37^	2001	Case–control	374	MSM GUM clinic attendees	Australia			X			X		
Valle^38^	1988	Cohort	235	MSM	Finland			X					
Gorbach et al.^39^	2019	Cohort	512	MSM	USA	X							
Barbee et al.^40^	2017	Case–control	880	MSM GUM clinic attendees	USA			X					
Down et al.^41^	2017	Cross‐sectional	545	MSM	Australia					X			
Wilkinson et al.^42^	2017	Cross‐sectional	4685	MSM	Australia			X					
Ferrer et al.^43^	2016	Cohort	3544	MSM GUM clinic attendees	Spain		X	X			X		X
Lyons et al.^44^	2014	Cohort	1034	MSM	Australia		X						
Templeton et al.^45^	2010	Cohort	1427	MSM	Australia		X						X
Menza et al.^46^	2009	Cohort	1903	MSM GUM clinic attendees	USA	X	X				X		X
Prestage et al.^47^	2009	Cross‐sectional	746	MSM	Australia		X						
Forna et al.^48^	2006	Case–control	132	Black women	USA			X					
McNulty et al.^49^	1997	Cohort	528	Sexually active MSM	Australia		X						
Craib et al.^50^	1995	Case–control	375	Gay men attending healthcare facilities	Canada	X	X						
Gattari et al.^51^	1994	Cohort	67	Prostitutes attending a GUM clinic	Italy						X		
Ellerbrock et al.^52^	1992	Cohort	1082	Pregnant women	USA		X						
Prestage et al.^53^	2009	Cohort	1427	MSM	Australia	X							
Thiede et al.^54^	2009	Cross‐sectional	142	MSM GUM clinic attendees	USA	X					X		X
Ellerbrock et al.^55^	2004	Cross‐sectional	1324	Resident of a rural community	USA		X	X					
Calzavara et al.^56^	2003	Cross‐sectional	183	MSM	Canada						X		X
Achterbergh et al.^57^	2020	Cross‐sectional	4461	MSM GUM clinic attendees	Netherlands	X							
Evers et al.^58^	2019	Cross‐sectional	600	MSM	Netherlands	X							
Drückler et al.^59^	2018	Cross‐sectional	4925	MSM GUM clinic attendees	Netherlands	X							
Pakianathan et al.^60^	2018	Cohort	1734	MSM GUM clinic attendees	England	X							
Tomkins et al.^61^	2018	Cohort	357	MSM GUM clinic attendees	England	X							
Bazan et al.^62^	2015	Cross‐sectional	331	Female GUM clinic attendees	USA	X							
Ottaway et al.^63^	2017	Case–control	260	MSM GUM clinic attendees	England	X					X		X
Heiligenberg et al.^64^	2012	Cross‐sectional	1861	MSM & female GUM clinic attendees	Netherlands	X							
Carey et al.^65^	2009	Case–control	444	MSM	USA	X		X			X		X
Niccolai et al.^66^	2004	Cross‐sectional	411	Adolescent women	USA					X			

### 3.2 Strength of evidence

Overall, previous bacterial STI, chemsex, number of recent partners, condom usage and type of sex had high scores and several key studies supported their link to acquisition risk (Table 4). One study linked SHS attendance to acquisition risk and very few studies, none of which were key studies, were found linking new sexual partners to risk. No studies were found linking partner exclusivity to acquisition risk.

**TABLE 4:** Results of scoring system for each sexual behaviour/indicator, number of keys studies associated with each sexual behaviour and the study populations key studies were carried out in.

Behaviour	Score	No. of key studies^a^	Study populations of key studies
Previous bacterial STI	49	9	GBMSM, SHS attendees, male sex workers
Chemsex	46	7	GBMSM
No. of recent partners	33	5	GBMSM
Condom use	28	5	GBMSM, trans women, SHS attendees
Type of sex	25	4	GBMSM, trans women
SHS attendance	3	1	SHS attendees
New sexual partner	3	0	N/A
Exclusivity	0	0	N/A

^a^ Many studies identified multiple behaviours associated with risk of HIV/STI acquisition, therefore this column adds up to more than the total number of key studies.

*Abbreviations*: GBMSM, gay, bisexual or other men who have sex with men; SHS, sexual health service.

### 3.3 Previous bacterial STI

The final score for this indicator was 49 and nine key studies supported a link between history of a bacterial STI and HIV/STI acquisition risk.^9^, ^12^, ^13^, ^14^, ^15^, ^17^, ^18^, ^19^, ^24^ Where key studies did not use baseline diagnosis as their metric but instead asked about STIs in a retrospective time period, all but one of them either used 6 or 12 months as their time period.^12^, ^14^, ^17^, ^18^, ^24^ Two of these retrospective studies looked at a history of any STIs and did not specify that the STI was bacterial.^12^, ^18^

Two cohort studies found participants who had a bacterial STI diagnosis (particularly syphilis, gonorrhoea or chlamydia) at the start of the study had an increased risk/rate of subsequent HIV acquisition, with one showing an HIV acquisition rate over three times higher for those diagnosed with syphilis; 7.2 (95% CI: 6.0–8.4) compared with 2.0 (95% CI: 1.7–2.3) diagnoses per 100 person years.^13^, ^14^ A third cohort study taking place in GBMSM attending SHS across England looked at the odds of developing a bacterial STI following their initial clinic attendance.^15^ It found those who had a bacterial STI at the start of the study had 1.43 (95% CI: 1.17–4.26) times the odds of acquiring a subsequent one.

### 3.4 Chemsex

The final score for this indicator was 46 and seven key studies supported a link between chemsex and HIV/STI acquisition risk.^12^, ^16^, ^17^, ^22^, ^23^, ^24^, ^25^ Some of the key studies did not specifically link drug use to sex but instead took use of drugs commonly used for chemsex, such as methamphetamine and gamma hydroxybutyrate (GHB)/gamma butyrolactone (GBL) as their risk factor.^16^, ^22^, ^25^ Within the key studies that investigated use of specific drugs, methamphetamine was most commonly linked to HIV/STI acquisition with GHB/GHL, mephedrone and amyl nitrate use also specifically being linked.

One cross‐sectional study by Sewell et al. and one cohort study by Koblin et al. looked at a range of drug use before/during sex, with the latter also including alcohol use.^17^, ^23^ They found that participants engaging in chemsex had 2.14 (95% CI: 1.83–2.50) times the odds of having had a bacterial STI and 1.58 (95% CI: 1.09–2.29) times the odds of HIV acquisition, respectively.

Several studies supported a link between chemsex and other potentially high‐risk behaviours, such as sex with a person who injects drugs (PWID), unprotected anal intercourse (UAI), higher numbers of sexual partners, group sex and lower condom use.^22^, ^23^, ^25^

### 3.5 Number of recent partners

The final score for this indicator was 33 and five key studies supported a link between increased numbers of partners and increased HIV/STI acquisition risk.^12^, ^15^, ^17^, ^18^, ^20^ One cohort study looked at high risk GBMSM across 47 US cities and in an adjusted regression model found that those with >10 partners in the past 6 months had 2.4 (95% CI: 1.7–3.3) times the odds of HIV acquisition compared with those with <5 partners.^12^ A cross‐sectional study in GBMSM in New Zealand also looked at number or partners in the past 6 months but with STI diagnosis as the outcome.^20^ It found significantly increased STI diagnosis for those with 6–10, 11–20, 21–50 and >50 partners compared with those with 1, and a general trend of increasing odds with increasing partner number was seen. One cross‐sectional study in GBMSM in London found increased odds of HIV acquisition up to 69.8 (95% CI: 35.5–138.2) with increasing numbers of sexual partners but this looked specifically at UAI, and so this is likely to explain the large odds ratios.^18^

### 3.6 Condom use

The final score for this indicator was 28 and five key studies found that decreased condom usage was associated with increased HIV/STI acquisition risk.^10^, ^11^, ^12^, ^17^, ^20^ Out of these only two specifically looked at condom usage not in combination with the type of sex. One was a cross‐sectional study of GBMSM in New Zealand which found that, compared with those reporting high usage, those reporting low/medium condom use with casual partners or with regular partners had increased odds of reporting a bacterial STI in the past year.^20^ The second was a case–control study on SHS attendees in Northern Italy, male and female, which found people reporting regular condom usage had 0.5 (95% CI: 0.4–0.5) times the odds of acquiring HIV compared with those reporting occasional or no use.^10^

Most of the key studies identified combined condom usage and type of sex, for example condomless anal sex, condomless receptive sex, or condomless receptive anal sex, and compared these groups to those not partaking in these behaviours.^11^, ^12^, ^17^ This makes it difficult to determine if the increased risk is associated with condom use, type of sex or both.

### 3.7 Type of sex

The final score for this indicator was 25 and four key studies showed an association between type of sex and HIV/STI acquisition risk.^11^, ^12^, ^17^, ^20^ One cross‐sectional study looked specifically at anal sex, regardless of insertive or receptive, and found that those reporting anal sex in the past 6 months had 2.3 (95% CI: 1.3–2.7) times the odds of reporting a bacterial STI in the past year.^20^

Other studies looked at anal sex in combination with condom usage which posed the same aforementioned issue with separating out which behaviour the risk is associated with.^11^, ^12^, ^17^ One such study found those having receptive UAI with a partner presumed to be negative had 1.92 (95% CI: 1.38–2.68) times the odds of HIV acquisition compared with those who did not engage in UAI.^17^ The other two studies found associations between HIV acquisition and condomless anal sex and between HIV acquisition and condomless receptive sex of any kind.^11^, ^12^ Overall, anal sex and specifically UAI the were found to be most associated with an increased risk although often these behaviours were not separated.

### 3.8 SHS attendance

The final score for this indicator was three and one key study showed an association between HIV/STI acquisition risk and attending a SHS.^21^ However, this cross‐sectional study did not compare SHS attendees to non‐attendees but rather took a population of attendees and looked at who opted out of HIV screening. It found that heterosexuals opting out had 1.85 (95% CI: 1.39–2.45) times the odds of having a history of STIs. Furthermore, it found that both heterosexuals and GBMSM who opted out had higher odds of having a current STI‐related complaint; 1.98 (95% CI: 1.57–2.51) and 4.22 (95% CI: 2.43–7.33), respectively.

A potential reason for very little evidence being found is because this behaviour meant restricting studies to those looking at screening within a healthcare setting rather than screening done elsewhere. For instance, a study by Ferrer et al.^24^ and another by Boyer et al.^67^ were not included because a healthcare setting was not specified. However, they found that those who had had an HIV test or STI check in the previous year had greater odds of testing positive for HIV or STIs at the time of the study, which suggests the opposite relationship to that suggested by the Dukers‐Muijrers et al.^21^ study.

### 3.9 New partners

The final score for this indicator was three and no key studies were identified that showed a link between having a new sexual partner and HIV/STI acquisition risk however, two articles were identified that did not meet the criteria to be classified as key studies.^41^, ^66^ One study in Australia found that few HIV infections occurred between GBMSM in long term sexual relationships and most occurred among GBMSM in new sexual relationships.^41^ The second study was among adolescent women in the US and found that having had a new sexual partner in the previous 12 months was strongly associated with STI acquisition.^66^

### 3.10 Exclusivity

The final score for this indicator was zero and no studies were found that linked exclusivity to HIV/STI acquisition. This is potentially because exclusivity was difficult to define or was defined by the study in terms of number of recent partners and so was categorised under this sexual behaviour instead. Some included studies did refer to casual sexual partners which could be interpreted as a substitute for non‐exclusive but was not interpreted as such in this review.

## 4 DISCUSSION

Our review found very strong evidence of a link between HIV/STI acquisition risk and having had a previous bacterial STI, having engaged in chemsex, and having had an increasing number of recent partners. These were therefore strongly recommended for consideration in a more individualised risk assessment for blood donors. These recommendations and the information from this study, alongside many other evidence pieces, helped to inform the wider FAIR review that occurred in 2021.

There was some consensus across studies when assessing recent bacterial STIs with most key studies looking at either a six or 12 month period since treatment completion being associated with an increased risk of further STIs or HIV.^9^, ^12^, ^13^, ^14^, ^15^, ^17^, ^18^, ^19^, ^24^ Since having had a bacterial STI represents an indicator of risk and not the source of risk itself then the time period used for this likely cannot be based on the commonly established period of 3 months for blood donor deferral. More research may help to determine an appropriate time period with a longer period representing a more cautious approach. With respect to chemsex, there was clear consensus that methamphetamine consumption increased risk but there was no clear consensus related to other chemsex drugs or what dosages related to acquisition risk.^12^, ^16^, ^17^, ^22^, ^23^, ^24^, ^25^ We therefore recommended further exploration of risk association with all drugs mentioned in the studies plus any other relevant drugs. Most literature found on chemsex had a study population of exclusively GBMSM which may mean this finding is less applicable to other groups, however some research suggests chemsex is more prevalent among GBMSM possibly minimising this issue of applicability.^68^ Furthermore, since the risk from chemsex comes from it's association with practices, such as condomless sex and high numbers of partners rather than chemsex itself it may be that the risk is mitigated from questions on other behaviours. We were not able to determine this from the evidence presented here. Given the strong evidence linking chemsex and acquisition risk a more cautious approach would be to include it alongside questions on other behaviours/indicators.

There was no unanimous agreement across the studies as to what number of recent partners during what time period is deemed as high risk. For blood donation purposes it may be pertinent to relate the time period applied for this to the established period of 3 months. Several studies found that acquisition risk increased with increasing number of sexual partners.^12^, ^18^, ^20^ More research is needed to identify the best cut off for number of recent partners but, in lieu of this, a more cautious approach of a low number may be optimal.

The strength of evidence for condom usage and for type of sex being linked to HIV/STI acquisition risk was strong but with potential limitations due to multiple studies combining the two behaviours thereby making it difficult to separate the effects of just one.^11^, ^12^, ^17^ It was suggested a question on either may be worth considering for inclusion in a more individualised risk assessment but further research would be required to untangle the risks posed by each. Furthermore, errors when using condoms and issues surrounding recall of condom use have previously been identified.^69^, ^70^, ^71^ While this was not looked at in this study it would potentially cause issues for questions surrounding condom use and so require consideration. Memory recall was evaluated elsewhere in the FAIR project. Looking at type of sex in reality encompasses several different behaviours, in particular: oral, vaginal or anal sex and receptive or penetrative sex. Many of the studies identified combined these, for example into receptive anal sex, sometimes specifying this was condomless too.^11^, ^12^, ^17^ However overall, there was a lot of agreement between studies that anal sex of various types, particularly receptive, represented the greatest risk and a question on this was also put forward for consideration in an individualised risk assessment.

There was very little evidence found to support SHS attendance being associated with HIV/STI acquisition risk.^21^ Testing for HIV/STIs in other settings was included in some studies we found but did not meet the criteria for consideration here. Those attending may represent a high‐risk group but they may also represent lower‐risk individuals who are conscious of mitigating risk. Therefore, including a question on SHS attendance in a more individualised risk assessment was not recommended, unless further research uncovered stronger evidence presenting a clear relationship with acquisition risk.

We found no evidence showing a relationship between exclusivity and HIV/STI acquisition. It is unlikely that no relationship exists, but rather that either the risk is so low it has not been studied or studies defined exclusivity in terms of numbers of sexual partners or ‘casual’ partners, which are not the same as exclusivity. This seems plausible given that many studies were found which looked at number of recent partners and several which referred to casual partners as defined by the participant. Having had a casual partner may be interpreted as a substitute for non‐exclusiveness, however, the opposite of a causal relationship is not necessarily an exclusive one and so conclusions about exclusivity cannot be drawn from these studies. We found very little evidence for a link between having had a new partner and acquisition. Given the nature of HIV/STI transmission and the strong evidence we identified linking number of partners with acquisition risk we believe this lack of evidence related to new partners is due to similar issues with definitions as with exclusivity. Our findings did not provide evidence for including exclusivity or new partners in a more individualised assessment however they should not be ruled out entirely given the issues with defining these terms in studies. Due consideration should be given to language understood by donors, which may differ from language used in research.

Based on our review alone there was insufficient evidence to support including a question on new sexual partners in a more individualised risk assessment.

For many of the behaviours/indicators looked at here the majority of studies relating to them relied on self‐reporting, in particular: chemsex, number of recent partners, condom use and type of sex. Exact methods varied between studies but self‐reporting is subject to recall bias so, although it is unclear the direction of effect this bias would have on these, this makes the findings in these less reliable. Self‐reporting is also required for blood donation screening and so reliability of recall is important when considering any of these for inclusion in a risk assessment and was specifically examined elsewhere for FAIR. While we applied exclusion criteria to limit study populations to those not excluded from donating due to other criteria it is likely many of the study populations still differ from typical donor populations. This was partly intentional as we wanted to look at populations wider than those eligible to donate prior to the 2021 rule change but nevertheless these differences may negatively impact the applicability of these studies to donor populations even after the rule change. On top of this the scoring system used here, while based on previously published work, is untested. This may produce bias in which studies are deemed to be more relevant and how evidence is weighted. However, the use of this system as a guide proved useful to draw conclusions but it is not advised that great emphasis is put on precise scores, which was avoided here. On top of this, studies involving PrEP/PEP users were excluded here which may call into question the applicability of our findings since these drugs are increasingly common among the UK population, especially GBMSM. However, currently individuals who have taken PrEP/PEP in the prior 3 months are prohibited from donating blood in the UK and, even if this were to change, the drugs are currently recommended for individuals at higher risk who would be likely be excluded for other reasons anyway. Further research into PrEP/PEP in relation to blood donation is being undertaken.

This review provided evidence for changes to blood donation policy in the UK and may be useful for other countries with similar epidemiology. It may also help to inform work outside the blood donation landscape such as in sexual health research/policy where defining high‐risk groups is important. However, when considering changes to blood donation policy this review must be considered in combination with research into reliability of recall, acceptability of screening questions and practical issues around implementation. These areas as they relate to the behaviours/indicators researched here were explored in other strands of the FAIR project.

## AUTHOR CONTRIBUTIONS

Conceptualization: K.L.D, S.R.B and J.F. Methodology: K.L.D, C.R. and J.F. Investigation: K.L.D. and J.F. Project administration: K.L.D, S.R.B and J.F. Writing—original draft: J.F. Writing—review & editing: K.L.D, S.R.B, C.R. and J.F.

## FUNDING INFORMATION

None.

## CONFLICT OF INTEREST STATEMENT

The authors have no competing interests.

## Supporting information


**Data S1.** Supporting Information.

## Data Availability

All data used comes from published literature, which is available to others. All literature included in our final analysis from which conclusions were drawn are included in the references.
