# Supplementary material for: Determining the strength of evidence for an association between sexual indicators and risk of acquiring HIV and sexually transmitted infections: Providing evidence for blood donation policy change
Source: Transfus Med. 2024 Jul 22;34(6):466–77. doi: 10.1111/tme.13062 (PMC11653059; doi:10.1111/tme.13062)
Supplement: Supplementary file 1 — Data S1. Supporting Information. [file TME-34-466-s001.docx]

**Supplementary material**

**Chemsex**

Database: Ovid Emcare <1995 to 2020 week 03>

Search Strategy:

--------------------------------------------------------------------------------

1 *sexually transmitted disease/ (4409)

2 *hepatitis B/ or *hepatitis C/ (12206)

3 *Human immunodeficiency virus/ (31043)

4 *syphilis/ (1814)

5 *gonorrhea/ (1137)

6 1 or 2 or 3 or 4 or 5 (49115)

7 (sex adj3 drug*).mp. [mp=title, abstract, heading word, drug trade name, original title, device manufacturer, drug manufacturer, device trade name, keyword] (1448)

8 chemsex.mp. (101)

9 slamsex.mp. (4)

10 7 or 8 or 9 (1534)

11 6 and 10 (440)

***************************

Database: Ovid MEDLINE(R) and Epub Ahead of Print, In-Process & Other Non-Indexed Citations, Daily and Versions(R) <1946 to January 23, 2020>

Search Strategy:

--------------------------------------------------------------------------------

1 *Sexually Transmitted Diseases/ (17233)

2 *Hepatitis C/ or *Hepatitis B/ (58904)

3 *HIV/ (11523)

4 *Syphilis/ (17863)

5 *Gonorrhea/ (10212)

6 *Sexually Transmitted Diseases, Bacterial/ (742)

7 "Sex on drugs".ti,ab. (2)

8 chemsex.ti,ab. (130)

9 "*HIV".kw. (38192)

10 "*STIs".kw. (118)

11 "*Syphilis".kw. (9498)

12 "*Chemsex".kw. (54)

13 *HIV Infections/di, ep, tm [Diagnosis, Epidemiology, Transmission] (36553)

14 1 or 2 or 3 or 4 or 5 or 6 or 9 or 10 or 11 or 13 (179188)

15 7 or 8 or 12 (143)

16 14 and 15 (71)

17 (sex adj3 drug*).mp. [mp=title, abstract, original title, name of substance word, subject heading word, floating sub-heading word, keyword heading word, organism supplementary concept word, protocol supplementary concept word, rare disease supplementary concept word, unique identifier, synonyms] (2948)

18 14 and 17 (801)

19 limit 18 to (humans and yr="1980 -Current") (719)

20 (HIV adj2 negative).mp. [mp=title, abstract, original title, name of substance word, subject heading word, floating sub-heading word, keyword heading word, organism supplementary concept word, protocol supplementary concept word, rare disease supplementary concept word, unique identifier, synonyms] (12874)

21 seronegative.mp. (17783)

22 20 or 21 (30052)

23 18 and 22 (55)

**GUM clinic attendance**

Database: Ovid Emcare <1995 to 2020 week 03>

Search Strategy:

--------------------------------------------------------------------------------

1 *Sexually Transmitted Diseases/ (1895)

2 *Hepatitis A/ or *Hepatitis B/ (5685)

3 *HIV Infections/ or *HIV/ (31036)

4 *Syphilis/ (1806)

5 *Gonorrhea/ (1130)

6 1 or 2 or 3 or 4 or 5 (40660)

7 "Genito-Urinary Medicine clinic*".ti,ab. (18)

8 "sexual health clinic*".kw. (11)

9 "*Sexual health clinics".kw. (5)

10 (Sexually transmitted disease* adj5 clinic).mp. [mp=title, abstract, heading word, drug trade name, original title, device manufacturer, drug manufacturer, device trade name, keyword] (399)

11 (Sexually transmitted disease* adj5 clinic).ti,ab. (396)

12 (STD adj5 clinic).ti,ab. (441)

13 (Sexually transmitted disease* adj5 screen*).ti,ab. (194)

14 (Sexually transmitted disease* adj5 test**).ti,ab. (246)

15 (STD adj5 screen*).ti,ab. (182)

16 (STD adj5 test*).ti,ab. (299)

17 (Sexually transmitted infection* adj5 clinic).ti,ab. (179)

18 (STI adj5 clinic*).ti,ab. (497)

19 (STIs adj5 screen*).ti,ab. (146)

20 11 or 12 or 13 or 14 or 15 or 16 or 17 or 18 or 19 (1973)

21 6 and 20 (774)

22 "HIV negative".ti,ab. (3666)

23 "HIV status".ti,ab. (4063)

24 "HIV seronegative ".ti,ab. (514)

25 sero-different.ti,ab. (6)

26 "HIV-uninfected partner*".ti,ab. (19)

27 "unknown hiv positive".ti,ab. (7)

28 22 or 23 or 24 or 25 or 26 or 27 (7478)

29 21 and 28 (40)

***************************

Database: Ovid MEDLINE(R) and Epub Ahead of Print, In-Process & Other Non-Indexed Citations and Daily <1946 to January 14, 2020>

Search Strategy:

--------------------------------------------------------------------------------

1 *Sexually Transmitted Diseases/ (17218)

2 *Hepatitis A/ or *Hepatitis B/ (46790)

3 *HIV Infections/ or *HIV/ (166334)

4 *Syphilis/ (17854)

5 *Gonorrhea/ (10202)

6 1 or 2 or 3 or 4 or 5 (250853)

7 "Genito-Urinary Medicine clinic*".ti,ab. (54)

8 "sexual health clinic*".kw. (13)

9 "*Sexual health clinics".kw. (0)

10 (Sexually transmitted disease* adj5 clinic).mp. [mp=title, abstract, original title, name of substance word, subject heading word, floating sub-heading word, keyword heading word, organism supplementary concept word, protocol supplementary concept word, rare disease supplementary concept word, unique identifier, synonyms] (1447)

11 (Sexually transmitted disease* adj5 clinic).ti,ab. (1444)

12 (STD adj5 clinic).ti,ab. (1363)

13 (Sexually transmitted disease* adj5 screen*).ti,ab. (402)

14 (Sexually transmitted disease* adj5 test**).ti,ab. (480)

15 (STD adj5 screen*).ti,ab. (379)

16 (STD adj5 test*).ti,ab. (577)

17 (Sexually transmitted infection* adj5 clinic).ti,ab. (393)

18 (STI adj5 clinic*).ti,ab. (1099)

19 (STIs adj5 screen*).ti,ab. (266)

20 11 or 12 or 13 or 14 or 15 or 16 or 17 or 18 or 19 (4965)

21 6 and 20 (2865)

22 "HIV negative".ti,ab. (11083)

23 "HIV status".ti,ab. (8287)

24 "HIV seronegative ".ti,ab. (2310)

25 sero-different.ti,ab. (8)

26 "HIV-uninfected partner*".ti,ab. (44)

27 "unknown hiv positive".ti,ab. (15)

28 22 or 23 or 24 or 25 or 26 or 27 (19830)

29 21 and 28 (177)

***************************

**Number of partners, new partners & exclusivity**

Database: Ovid Emcare <2015 to 2020 week 03>

Search Strategy:

--------------------------------------------------------------------------------

1 *Human immunodeficiency virus/ (5441)

2 *sexually transmitted disease/ (1277)

3 *hepatitis C/ (2571)

4 *hepatitis B/ (1308)

5 *syphilis/ (634)

6 *gonorrhea/ (464)

7 *Human immunodeficiency virus infection/ (10537)

8 1 or 2 or 3 or 4 or 5 or 6 or 7 (20441)

9 exclusivity.ti,ab. (325)

10 *monogamy/ (40)

11 *polygamy/ (11)

12 polygamous.mp. (52)

13 "anonymous partner*".ti,ab. (8)

14 "new sex* partner*".ti,ab. (33)

15 (number adj2 "sex partner*").mp. [mp=title, abstract, heading word, drug trade name, original title, device manufacturer, drug manufacturer, device trade name, keyword] (54)

16 "sexually exclusive".ti,ab. (0)

17 9 or 10 or 11 or 12 or 13 or 14 or 15 or 16 (509)

18 "HIV negative".ti,ab. (1421)

19 "HIV status".ti,ab. (1628)

20 "HIV seronegative ".ti,ab. (93)

21 sero-different.ti,ab. (4)

22 "HIV-uninfected partner*".ti,ab. (9)

23 18 or 19 or 20 or 21 or 22 (2869)

24 17 and 23 (18)

***************************

Database: Ovid MEDLINE(R) and Epub Ahead of Print, In-Process & Other Non-Indexed Citations and Daily <1946 to January 14, 2020>

Search Strategy:

--------------------------------------------------------------------------------

1 *Sexually Transmitted Diseases/ (17218)

2 *Hepatitis C/ or *Hepatitis B/ (58861)

3 *HIV Infections/ or *HIV/ (166334)

4 *Syphilis/ (17854)

5 *Gonorrhea/ (10202)

6 1 or 2 or 3 or 4 or 5 (259981)

7 (number adj2 "sex partner*").mp. [mp=title, abstract, original title, name of substance word, subject heading word, floating sub-heading word, keyword heading word, organism supplementary concept word, protocol supplementary concept word, rare disease supplementary concept word, unique identifier, synonyms] (572)

8 exclusivity.ti,ab. (1810)

9 *Sexual Partners/ (7348)

10 "anonymous partner*".ti,ab. (67)

11 (sexual adj2 partners).mp. [mp=title, abstract, original title, name of substance word, subject heading word, floating sub-heading word, keyword heading word, organism supplementary concept word, protocol supplementary concept word, rare disease supplementary concept word, unique identifier, synonyms] (22934)

12 "sexually exclusive".ti,ab. (9)

13 monogamy.ti,ab. (930)

14 polygamy.ti,ab. (373)

15 polygamous.ti,ab. (524)

16 7 or 8 or 9 or 10 or 11 or 12 or 13 or 14 or 15 (26524)

17 6 and 16 (9204)

18 17 (9204)

19 limit 18 to (humans and yr="1980 -Current") (9019)

20 "HIV negative".ti,ab. (11083)

21 "HIV status".ti,ab. (8287)

22 "HIV seronegative ".ti,ab. (2310)

23 sero-different.ti,ab. (8)

24 20 or 21 or 22 or 23 (19786)

25 17 and 24 (1355)

26 25 (1355)

27 limit 26 to (humans and yr="1980 -Current") (1351)

28 from 27 keep 1-3,5,7,11,13,15-16,18,20,23,26,28-1351 (1337)

***************************

**Condom use & type of sex**

Database: Ovid Emcare <2015 to 2020 week 03>

Search Strategy:

--------------------------------------------------------------------------------

1 *Human immunodeficiency virus/ (5441)

2 *sexually transmitted disease/ (1277)

3 *hepatitis C/ (2571)

4 *hepatitis B/ (1308)

5 *syphilis/ (634)

6 *gonorrhea/ (464)

7 *Human immunodeficiency virus infection/ (10537)

8 1 or 2 or 3 or 4 or 5 or 6 or 7 (20441)

9 "HIV negative".ti,ab. (1421)

10 "HIV status".ti,ab. (1628)

11 "HIV seronegative ".ti,ab. (93)

12 sero-different.ti,ab. (4)

13 "HIV-uninfected partner*".ti,ab. (9)

14 9 or 10 or 11 or 12 or 13 (2869)

15 "condom adj2 usage".ti,ab. (0)

16 "condom usage".ti,ab. (35)

17 *"condom use"/ (516)

18 (oral sex adj5 only).ti,ab. (5)

19 *"oral sex"/ (35)

20 *safe sex/ (100)

21 *sexual behavior/ (1576)

22 15 or 16 or 17 or 18 or 19 or 20 or 21 (2194)

23 8 and 22 (577)

24 14 and 23 (92)

***************************

Database: Ovid MEDLINE(R) and Epub Ahead of Print, In-Process & Other Non-Indexed Citations, Daily and Versions(R) <1946 to January 14, 2020>

Search Strategy:

--------------------------------------------------------------------------------

1 *Human immunodeficiency virus/ (11517)

2 *sexually transmitted disease/ (17220)

3 *hepatitis C/ (31432)

4 *hepatitis B/ (31747)

5 *syphilis/ (17855)

6 *gonorrhea/ (10203)

7 *HIV/ (11517)

8 1 or 2 or 3 or 4 or 5 or 6 or 7 (113724)

9 "HIV negative".ti,ab. (11085)

10 "HIV status".ti,ab. (8287)

11 "HIV seronegative ".ti,ab. (2310)

12 sero-different.ti,ab. (8)

13 "HIV-uninfected partner*".ti,ab. (44)

14 9 or 10 or 11 or 12 or 13 (19821)

15 "condom adj2 use".ti,ab. (0)

16 "condom usage".ti,ab. (305)

17 (oral sex adj5 only).ti,ab. (46)

18 *"oral sex"/ (30791)

19 *safe sex/ (1281)

20 *sexual behavior/ (30791)

21 "condom use".ti,ab. (10033)

22 15 or 16 or 17 or 18 or 19 or 20 or 21 (38642)

23 8 and 14 and 22 (147)

***************************

**General**

Database: Ovid Emcare <1995 to 2020 week 03>

Search Strategy:

--------------------------------------------------------------------------------

1 *Sexually Transmitted Diseases/ (1895)

2 *Hepatitis A/ or *Hepatitis B/ (5685)

3 *HIV Infections/ or *HIV/ (31036)

4 *Syphilis/ (1806)

5 *Gonorrhea/ (1130)

6 1 or 2 or 3 or 4 or 5 (40660)

7 "HIV negative".ti,ab. (3666)

8 "HIV status".ti,ab. (4063)

9 "HIV seronegative ".ti,ab. (514)

10 sero-different.ti,ab. (6)

11 "HIV-uninfected partner*".ti,ab. (19)

12 "unknown hiv positive".ti,ab. (7)

13 7 or 8 or 9 or 10 or 11 or 12 (7478)

14 *Risk-Taking/ (3070)

15 "HIV infection risk*".ti,ab. (122)

16 *Risk Factors/ (21192)

17 *risk/ (45068)

18 14 or 15 or 16 or 17 (67461)

19 6 and 18 (3514)

20 13 and 19 (363)

***************************

Database: Ovid MEDLINE(R) and Epub Ahead of Print, In-Process & Other Non-Indexed Citations, Daily and Versions(R) <1946 to January 14, 2020>

Search Strategy:

--------------------------------------------------------------------------------

1 *Sexually Transmitted Diseases/ (17220)

2 *Hepatitis A/ or *Hepatitis B/ (46790)

3 *HIV Infections/ or *HIV/ (166340)

4 *Syphilis/ (17855)

5 *Gonorrhea/ (10203)

6 1 or 2 or 3 or 4 or 5 (250862)

7 "HIV negative".ti,ab. (11085)

8 "HIV status".ti,ab. (8287)

9 "HIV seronegative ".ti,ab. (2310)

10 sero-different.ti,ab. (8)

11 "HIV-uninfected partner*".ti,ab. (44)

12 "unknown hiv positive".ti,ab. (15)

13 7 or 8 or 9 or 10 or 11 or 12 (19832)

14 *Risk-Taking/ (11572)

15 "HIV infection risk*".ti,ab. (273)

16 *Risk Factors/ (1116)

17 *risk/ (4032)

18 14 or 15 or 16 or 17 (16931)

19 6 and 18 (3342)

20 13 and 19 (334)

21 from 20 keep 1-2,4,6-8,10-17,20-21,23-24,30-42,44-54,56-65,69-70,73-76,78,81-83,85,87-99,103-104,106-110,112-115,117-118,121,123,125-129,131,134-135,141-143,145,147-154,156-157,160-162,165-166,169,171-176,178-179,184-186,188-189,191-193,197,201-202,205-207,209,211-212,214-217,219,222-224,226-228,231-239,241,246-248,250-251,253-255,258,260-267,269-282,284-288,290-313,315,317-318,320-323,325-329,331-332,334 (240)

***************************
